# Cytotoxicity of Light-Cured Dental Materials according to Different Sample Preparation Methods

**DOI:** 10.3390/ma10030288

**Published:** 2017-03-14

**Authors:** Myung-Jin Lee, Mi-Joo Kim, Jae-Sung Kwon, Sang-Bae Lee, Kwang-Mahn Kim

**Affiliations:** 1 Department and Research Institute of Dental Biomaterials and Bioengineering, Yonsei University College of Dentistry, Seoul 03722, Korea; lmj239@yuhs.ac (M.-J.L.); MIJOOKIM@yuhs.ac (M.-J.K.); JKWON@yuhs.ac (J.-S.K.); 2 Brain Korea 21 PLUS Project, Yonsei University College of Dentistry, Seoul 03722, Korea; 3 Dental Device Testing and Evaluation Center, Yonsei University College of Dentistry, Seoul 03722, Korea

**Keywords:** biocompatibility, cytotoxicity, oxygen-inhibition layer, sample preparation, resin-based materials

## Abstract

Dental light-cured resins can undergo different degrees of polymerization when applied in vivo. When polymerization is incomplete, toxic monomers may be released into the oral cavity. The present study assessed the cytotoxicity of different materials, using sample preparation methods that mirror clinical conditions. Composite and bonding resins were used and divided into four groups according to sample preparation method: uncured; directly cured samples, which were cured after being placed on solidified agar; post-cured samples were polymerized before being placed on agar; and “removed unreacted layer” samples had their oxygen-inhibition layer removed after polymerization. Cytotoxicity was evaluated using an agar diffusion test, MTT assay, and confocal microscopy. Uncured samples were the most cytotoxic, while removed unreacted layer samples were the least cytotoxic (*p* < 0.05). In the MTT assay, cell viability increased significantly in every group as the concentration of the extracts decreased (*p* < 0.05). Extracts from post-cured and removed unreacted layer samples of bonding resin were less toxic than post-cured and removed unreacted layer samples of composite resin. Removal of the oxygen-inhibition layer resulted in the lowest cytotoxicity. Clinicians should remove unreacted monomers on the resin surface immediately after restoring teeth with light-curing resin to improve the restoration biocompatibility.

## 1. Introduction

Resin-based dental materials are widely used in restorative dentistry, owing to their many desirable qualities, including excellent esthetic outcome, easy handling, favorable mechanical properties, and improved bonding efficiency [1,2,3]. However, concerns remain over the biocompatibility of unreacted resin monomers following incomplete polymerization of such light-cured materials [4,5,6]. Extracts from resin composites have been reported to have genotoxic, mutagenic, and estrogenic effects [7,8,9,10,11]. Therefore, toxicity levels need to be experimentally determined to clarify the safety of resin-based dental materials in clinical settings [7,8,12,13].

There have been contradictory reports concerning the biocompatibility of resin-based materials. Ruey-Song Chen et al. [14] reported the cytotoxicity of three dentin bonding agents on human dental pulp cells. In contrast, Alexander Franz et al. [15] reported no significant cytotoxic effects of dental bonding substances on L929 cells. Another study tested the same resin-based materials and reported very different results [16]. However, these studies differed in sample preparation methods, cell lines, application methods, and types of products. Perhaps most importantly, different products undergo different degrees of conversion and, thus, produce different amounts of monomer extract, thereby leading to large variations in cytotoxicity [10,17,18,19]. Therefore, standardized sample preparation and curing procedures are necessary to assess the actual toxicity of dental resin-based materials.

The international standard ISO 7405, which addresses the biocompatibility of dental materials and devices, states that biocompatibility tests should be performed on materials in an “as-used state.” In the case of light-curing materials, the standards recommend that light curing be performed in the presence of oxygen, reflecting the conditions that are experienced in clinical use [20]. According to the recommendations, light-curable composites should be polymerized using a light-curing unit for 20 s and then used in biocompatibility tests. However, complete curing is not always possible in clinical practice because of the existence of saliva or anatomical problems [21,22]. The incomplete curing thus leads to the release of cytotoxic leachable monomers. Hence, toxicity should be tested in vitro and in vivo to elucidate the actual effects of dental materials.

Accordingly, in the present study, we evaluated the cytotoxicity of two types of light-curing resin under four different conditions, including uncured, direct light-cured, post-cured, and post-cured samples, with the removal of the oxygen-inhibition layer. We aimed to assess the cytotoxicity of resin-based dental materials under conditions that closely mimic those encountered in clinical practice.

## 2. Materials and Methods

### 2.1. Test Cells

L929 mouse fibroblasts (Korean Cell Line Bank, Korea) were cultivated in RPMI 1640 (Sigma, Irvine, CA, UK) containing 10% fetal bovine serum (FBS; Gibco, Grand Island, NY, USA) and 1% penicillin/streptomycin (Invitrogen, Grand Island, NY, USA) in a humidified incubator with 5% CO_2_ at 37 °C. Confluent cells were detached using 0.25% trypsin (Sigma, St. Louis, MO, USA), and aliquots of separated cells were sub-cultured. Cells between the 7th and 14th passages were used for the experimental procedures.

### 2.2. Test Materials and Sample Preparations

**Figure 1:**
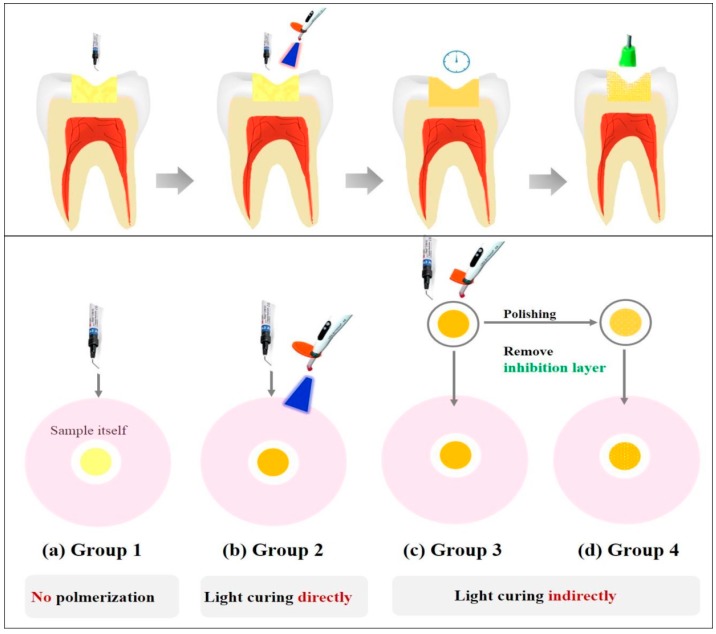
A representative illustration of the different sample preparation methods. (**a**) Samples were used without any curing; (**b**) samples were cured on agar with a light curing unit for 20 s; (**c**) samples were used after polymerization; (**d**) samples were polymerized and the unreacted resin monomer layer was removed afterward.

Two types of dental light-curing materials, composite resin (C groups; 3M ESPE Filtek™ Z350XT, St. Paul, MN, USA) and bonding resin (B groups; 3M ESPE Adper Scotchbond™, St. Paul, MN, USA), were used in the present study (Table 1); the selection of test materials was based on the previous studies [2,15,17]. The test specimens were prepared to a thickness of 2 mm and a diameter of 5 mm in a Teflon mold. They were sterilized using ethylene oxide gas treatment. In consideration of clinical process of using the materials, these were divided into four different conditions of sample preparation, as follows (Table 2): group 1—uncured (CU, BU) samples were used without being polymerized; group 2—direct light cured (CD, BD) samples were placed on the solidified agar without being polymerized and cured directly using a light curing unit (3M Elipar Free Light 2, St. Paul, MN, USA, 650 mW/cm^2^) for 20 s at a distance of 3 mm from the samples according to manufacturer’s instructions; group 3—post-cured (CP, BP) samples were used after being polymerized; group 4—“removed unreacted layer” (CR, BR) samples were polymerized using a translucent polyethylene film to protect the resin materials from oxygen exposure. Following sample preparation, the samples were polished using #1500-grit silicon carbide paper for 15 s. A schematic illustration of the sample preparation methods is shown in Figure 1.

### 2.3. Degree of Conversion (DC)

The measurements of the DC (*n* = 5) were evaluated by Fourier Transform Infrared Spectroscopy (FTIR; Vertex 70, Bruker Optik, Ettlingen, Germany). The spectrometer was coupled to a horizontal attenuated total reflectance (ATR) device consisting of a diamond crystal 2 mm in diameter (Platinum ATR-QL, Bruker Optik, Baden-Württemberg, Germany). The diameter of the measured surface was 800 µm, the wave number range of the spectrum was 2000–1400 cm^−1^ and the FTIR spectra were recorded with two scans/s at a resolution of 4 cm^−1^. To determine the percentage of the remained unreacted double bonds, the DC was assessed as the variation of the absorbance intensities peak area ratio of the methacrylate car-bon double bond (peak 1634 cm^−1^) and those of an internal standard (aromatic carbon double bond; peak at 1608 cm^−1^) during polymerization, in relation to the uncured material [23]:

(1) $${\mathrm{DC}}_{\mathrm{Peak}\;\mathrm{height}}(\%)=\left(1-\frac{\left(1634\;{\mathrm{cm}}^{-1}/1608\;{\mathrm{cm}}^{-1}\right)\mathrm{Peak}\;\mathrm{height}\;\mathrm{after}\;\mathrm{curing}}{\left(1634\;{\mathrm{cm}}^{-1}/1608\;{\mathrm{cm}}^{-1}\right)\mathrm{Peak}\;\mathrm{height}\;\mathrm{before}\;\mathrm{curing}}\right)\times 100$$

### 2.4. Eluent Preparation

Preparation of extracts from the samples was performed in accordance with international standards [24]. Because groups 2 and 3 had the same eluents, the extracts were divided as follows: “Uncured”, “Post-cured”, and “Removed unreacted layer”. Despite the limitation of extraction for hydrophobic material used in this study, choice of eluent was based on previous studies [2,8,18] and consideration of cellular experiment. The appropriate amount of each sample was added to a culture medium (RPMI 1640; Gibco, Grand Island, NY, USA) supplemented with 10% FBS inside a sterilized glass bottle. The extraction ratio was 0.2 g/mL, according to ISO 10993-12. The samples and extraction solution were incubated at 37 °C in a humidified atmosphere of 5% CO_2_ for 24 h. After incubation, the culture medium containing material extracts was filtered through 0.22 µm cellulose acetate filters (Millipore, Sigma, St. Louis, MO, USA) and the extracts were used for the cytotoxicity testing in the MTT assay.

### 2.5. Agar Diffusion Test

This test was performed to evaluate the nonspecific cytotoxicity of test materials after diffusion through agar according to ISO 10993-5 [24]. Cell suspensions (2.5 × 10^5^ cells/mL) in a 10 mL volume were seeded in 100 mm diameter cell culture dishes (SPL, Pocheon-Si, Gyeonggi-Do, Korea) and incubated at 37 °C in a humidified atmosphere with 5% CO_2_. After 24 h, the medium was replaced with 10 mL of freshly prepared agar medium containing 2× RPMI 1640 (Sigma, Irvine, Ayrshire, UK). Following solidification of the liquid culture medium, 10 mL of neutral red solution (0.01% in phosphate-buffered saline, Sigma, St. Louis, MO, USA) was added in the dark for 20 min. Excess neutral red solution was aspirated, and the test specimens were placed on the agar surface along with the positive (latex sheet) and negative controls (Teflon mold) in the same cell culture dish. 

After 24 h of incubation, the decolorization index and lysis index were assessed using an optical microscope according to ISO 7405 [20]. The decolorized zones were scored as follows: 0 = no decolorization detectable; 1 = decolorization only under the specimen; 2 = decolorization in a zone not greater than 5 mm from the specimen; 3 = decolorization in a zone not greater than 10 mm from the specimen; 4 = decolorization in a zone greater than 10 mm from the specimen; 5 = the total culture is decolorized. Cell lysis was defined as a loss of cell membrane integrity, visible under light microscopy. Cell lysis was scored as follows: 0 = no cell lysis detectable; 1 = less than 20% cell lysis; 2 = 20%–40% cell lysis; 3 = 40%–60% cell lysis; 4 = 60%–80% cell lysis; 5 = greater than 80% cell lysis. The test was performed in quadruplicate.

### 2.6. MTT Assay

The MTT assay is a colorimetric assay used to measure cell viability. Yellow water-soluble MTT is metabolically reduced in viable cells to a blue-violet insoluble formazan. This test was performed in accordance with ISO 10993-5 [24]. The L929 cells were seeded at a density of 1 × 10^5^ cells/mL into 96-well plates (SPL, Pocheon-Si, Gyeonggi-Do, Korea) and maintained in culture for 24 h to form a semi-confluent monolayer. The culture medium was removed from the wells, and 100% extractions from samples or serial dilutions of extractions using culture medium (50%, 25%, 12.5%, and 6.25%) in a 100 µL volume were placed into each well. After 24 h, the culture medium was removed and replaced with 50 µL of MTT solution in phosphate-buffered saline (PBS, 1 mg/mL). The MTT solution was then discarded and 100 µL of isopropanol was added to each well. The plates were shaken until all crystals were dissolved. The absorbance was spectrophotometrically measured using an ELISA reader (Epoch, BioTek, Winooski, VT, USA) at 570 nm. The assay was conducted in triplicate.

### 2.7. Cytotoxicity Evaluation

Cytotoxicity was assessed by exposing 100% of each extraction type of composite resins and bonding resins to the cells for 24 h, followed by staining with calcein AM and ethidium homodimer-1 (Molecular Probes, Eugene, OR, USA) for observation under a confocal laser microscope (LSM700, Carl Zeiss, Thornwood, NY, USA). Live cells were stained to produce a green fluorescence, and bright red fluorescence was observed from dead cells. Live and dead cells were quantified according to normalized surface areas of staining intensity using ImageJ software.

### 2.8. Statistics

Statistical analysis was performed using one-way analysis of variance (ANOVA) and the independent *t*-test (equal variance not assumed); *p* ≤ 0.05 were considered to be statistically significant. SPSS PASW version 21.0 (SPSS Inc., Chicago, IL, USA) was used for statistical analysis.

## 3. Results

### 3.1. Degree of Conversion (DC)

Both materials showed significant differences in degree of conversion according to the method of sample preparation, as indicated in Table 3.

### 3.2. Agar Diffusion Test

**Figure 2:**
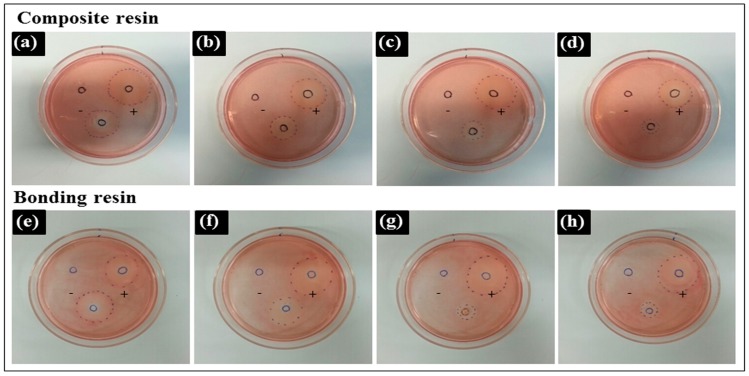
Decolorization zones of experimental materials in the agar diffusion test. The empty Teflon mold (negative control), the latex sheet from the latex glove (positive control), and experimental samples of (**a**) uncured composite resin (CU); (**b**) directly cured composite resin (CD); (**c**) post-cured composite resin (CP); (**d**) composite resin after removing the unreacted layer (CR); (**e**) uncured bonding resin (BU); (**f**) directly cured bonding resin (BD); (**g**) post-cured bonding resin (BP); (**h**) bonding resin after removing the unreacted layer (BR) were located in predetermined positions. Representative images are shown after experiments were performed in triplicate.

The decolorization zones of the experimental materials are presented in Figure 2.

A Teflon mold was used as a negative control, which caused no cellular lysis, whereas the latex sheet, a positive control, resulted in a cytotoxicity score of 4. The cytotoxicity scores of the various preparations are shown in Table 4. Uncured resins (CU, BU) had the highest cytotoxicity. Samples with the oxygen-inhibition layer removed (CR, BR) showed lower cytotoxicity than post-cured samples (CP, BP). There was no significant difference between the cytotoxicity of composite resin and bonding resin.

### 3.3. MTT Assay

**Figure 3:**
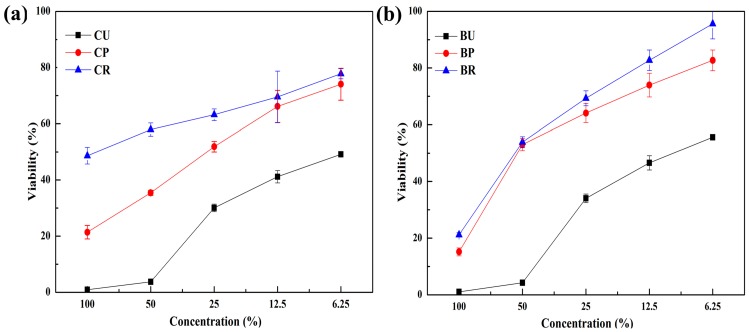
Cell viability following exposure to the extracts from (**a**) composite resin and (**b**) bonding resin at different dilutions. B: bonding resin; C: composite resin; U: uncured; P: post-cured; R: unreacted layer removed.

The results of the cell viability assay are shown in Figure 3. The uncured samples (CU, BU) had a significantly higher cytotoxicity than any others, regardless of sample preparation methods. The mean (±SD) cell viabilities for undiluted CU, CP, and CR were, 0.89% ± 0.83%, 21.40% ± 2.39%, and 48.67% ± 2.96%, respectively. A similar trend was found in BU, BP, and BR (1.01% ± 0.08%, 15.15% ± 1.41% and 21.18% ± 0.71%, respectively). There was no significant difference in the viabilities by CP and CR, which were 12.5% and 6.25%, respectively (*p* > 0.05). The viability increased in the order of CU < CP < CR in the composite resin groups, and this was the same in the bonding resin groups, i.e., BU < BP < BR.

**Figure 4:**
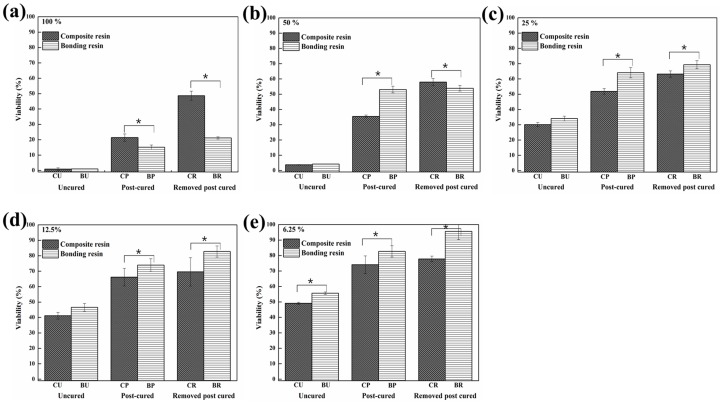
Cell viability following exposure to the extracts from the composite resin and bonding resin at different dilutions: (**a**) 100% concentration; (**b**) 50% concentration; (**c**) 25% concentration; (**d**) 12.5% concentration; (**e**) 6.25% concentration (*: Statistically significant at *p* < 0.05).

The cell viabilities according to composite and bonding resin in each dilution are shown in Figure 4. The viability by BP and BR was significantly higher than that by CP and CR, respectively (*p* < 0.05), except for the post-cured and “Removed unreacted layer” groups at a 100% concentration and the “Removed unreacted layer” group at 50% concentration.

### 3.4. Live/Dead Image Assay^®^

**Figure 5:**
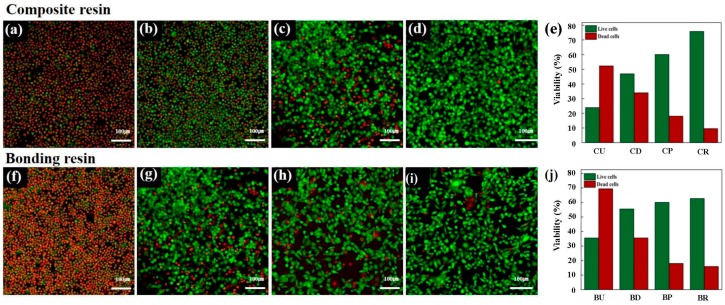
Confocal laser microscopy images following calcein AM and ethidium homodimer-1 staining of L929 cells. Cells were exposed to extracts from (**a**) uncured composite resin; (**b**) directly cured composite resin; (**c**) post-cured composite resin; (**d**) composite resin after removing the unreacted layer; (**e**) Live/Dead Assay® quantified the live and dead cells in equivalent surface areas of composite resin; (**f**) uncured bonding resin; (**g**) directly cured bonding resin; (**h**) post-cured bonding resin; (**i**) bonding resin after removing the unreacted layer; (**j**) Live/Dead Assay^®^ quantified the live and dead cells in equivalent surface areas of bonding resin. Live cells are stained green and dead cells are stained red for confocal laser microscope images.

The cytotoxicity results were also confirmed by staining cells with calcein AM and ethidium homodimer-1 (Molecular Probes, Eugene, OR, USA) for observation under a confocal laser microscope (LSM700M, Carl Zeiss, Thornwood, NY, USA). Intense green fluorescence was observed from live cells and red fluorescence was observed from dead cells (Figure 5). The results from the Live/Dead Assay^®^ image assay were in an agreement with the results of the agar diffusion test and MTT assay. 

## 4. Discussion

A standardized protocol for biocompatibility evaluation is essential to assess the safety of dental materials. Several studies have investigated the cytotoxicity of resin-based materials and found that unreacted dental resin monomers are toxic to human gingival fibroblasts and keratinocytes. These monomers can be released from the final product, which can also be cytotoxic itself, according to in vitro studies [6,25]. Geurtsen et al. [9] reported that the bisphenol-A-glycidyl methacrylate (Bis-GMA) monomer has the strongest cytotoxicity, followed sequentially by UDMA, TEGDMA, and HEMA. In addition, all resin monomers exhibited a dose-dependent genotoxicity [7,14,26,27,28]. Furthermore, it has been reported that unreacted resin-based materials may release substances into an aqueous environment for extended periods, possibly causing cell damage and pulpal inflammation [8,18]. These findings suggest that a cytotoxicity test is suitable for the evaluation of basic biocompatibility [24]. 

There are numerous techniques available for cytotoxicity evaluation, such as the MTT assay, agar diffusion test, filter diffusion test, and pulp and dentine usage test [20,24,29,30]. Two test methods were used in this study: the agar diffusion test and MTT assay. In the agar diffusion test, samples were separated from the cells by an agar layer mimicking the mucosal membrane, whereas in the MTT assay, extracts of the samples were used, mimicking constituents leaching into the saliva. The endpoint of the agar diffusion test is membrane integrity, and that of the MTT assay is mitochondrial activity. The methods address different aspects of cytotoxicity, and the results collectively provide an overview of the cytotoxic potential of the samples [31]. Previous in vitro studies have yielded contradictory findings regarding the cytotoxicity of resin-based dental materials [6,16,27,31,32]. These differences likely arise because in vitro cytotoxicity tests do not accurately reflect the clinical situation. In the clinic, resin-based materials are inserted into the oral cavity in a freshly mixed, incompletely polymerized stage; local responses are provoked by unreacted or only partially reacted components. After polymerization, the surface is usually polished to remove the oxygen inhibition layer, which may cause the release of toxic constituents from the material [8]. To optimize cytotoxicity evaluation, suitable sample preparation methods are important. In this study, the effect of these different conditions on the sample preparation of resin based materials was investigated. We applied the various conditions shown in Table 2. The results showed similar trends in both the agar diffusion and MTT tests; however, there was a significant difference in cell viability according to different sample preparation methods for the same materials. This was also confirmed in the images obtained using confocal laser microscopy. Uncured samples had the highest cytotoxicity, while the samples retaining the oxygen-inhibition layer had a higher degree of toxicity than those that underwent the polishing process. In this respect, the removal of the inhibition layer was found to be a crucial factor for increased cell viability.

In this study, the degree of conversion was assessed using FTIR spectroscopy (Table 3). The degree of conversion would indicate the proportion of polymerized products from their original monomer state through free radical polymerization reactions. In other words, the lower the degree of conversion, greater the proportion of unpolymerized monomers available to cells during the cytotoxicity tests. The results indicated that the value for the degree of conversion was approximately 75% and 49% for direct-cured composite resin and adhesive resin, respectively. The values then increased as post-cured samples were analyzed (approximately 88% and 61% for composite resin and adhesive resin, respectively). These values were, in fact, in agreement with previous studies that used either the same or similar products [33,34]. However, the degree of conversion further increased to approximately 95% for both products after removal of the oxygen inhibition layer, which is an extremely high value―such a result has not been previously reported in the literature. It was clear that the degree of conversion of the two materials, in descending order, was samples with unreacted layer removed > post-cured > directly cured > uncured.

The results were then compared with the cytotoxicity tests. The agar diffusion test indicated that both directly cured and uncured samples were more cytotoxic than both post-cured and those with the unreacted layer removed. Additionally, Figure 3 and Figure 4 indicated that the order of cell viability following MTT assay was unreacted layer removed > post-cured > directly cured > uncured, for both bonding resin and composite resin―an order equivalent to the degree of conversion. This finding was further confirmed by fluorescence imaging in the Live/Dead Assay^®^. It is well known that oxygen inhibits free radical polymerization and yields polymers with uncured surfaces [35]. Hence, it may be the reason for the extremely high degree of conversion value for samples with the unreacted layer removed. Removal of such a layer would have increased the degree of conversion and, consequently, the adequate polymerization would have influenced the biocompatibility of the restoration [36].

As shown in Figure 3 and Figure 4, there was a difference in cytotoxicity between composite and bonding resins. Generally, composite resin has a higher filler content than bonding resin, which contains water, alcohol, or acetone, although the contents differ depending on the product [19]. These hydrophilic components may affect the solubility of bonding resin, i.e., cells may be more easily affected by toxic materials from bonding resins. However, as the dilutions increase, the effects of hydrophilic resin monomers, such as Bis-GMA, UDMA, TEGDMA or camphorquinone, have a critical impact on total cell viability in the MTT assay. It has also been reported that filler contents appear to influence polymerization [37]. The results shown in Figure 3 and Figure 4 can be explained by these differences in released components and filler contents.

Although the MTT assay revealed differences between dilutions and resin types, these differences were not observed in the agar diffusion test. Because an agar overlay test only quantitatively demonstrates the decolorization zone and lysis index, cytotoxicity is usually confirmed by other qualitative methods such as an MTT assay. 

This study showed that different methods of sample preparation led to different cytotoxicity levels of dental resin. Because the present study was conducted on mouse fibroblast cells, as recommended by international standards, conclusions regarding the possible toxicity in vivo are limited [24]. A major concern regarding in vitro test data is the relatively poor correlation among these tests. The International Standards Organization (ISO) recommends the use of established cell lines, such as L929 mouse fibroblasts, for cytotoxicity tests. Because L929 cells are easy to prepare and culture, they are commonly used for cell culture-based standardization of cytotoxicity studies [20,24]. In addition, L929 cells are highly sensitive to the lytic action of cytotoxins, and exhibit a greater decrease in cell viability than other cell lines [31,38]. This enables greater sensitivity in assessing the degree of cytotoxicity.

Dental materials, such as resin composites and bonding agents, can harm teeth and the surrounding soft tissues, and lead to hypersensitivity or other symptoms when applied clinically. Therefore, tests methods that mimic in vivo conditions, such as a dentin barrier test will, no doubt, be more clinically relevant. Although our protocol does not reflect clinical conditions as well as an extended dentin barrier test or a long-term in vivo study, it is economical and easily available. Further studies comparing and correlating cytotoxicity results with a dentin barrier test or in vivo test will produce more clinically relevant results. Despite these limitations, the present study indicated that the selection of an improper method may lead to false-negative cytotoxicity results. Hence, careful consideration in selecting the sample preparation is required.

Furthermore, our findings may have implications for the selection of sample preparation method. More specifically, clinicians should remove unreacted monomers on the resin surface immediately after restoring teeth with light-curing resin to limit cytotoxic effects.

## 5. Conclusions

The cytotoxicity of resin-based dental materials depends on the sample preparation method. Uncured materials were the most cytotoxic, followed by light-cured materials and those with the oxygen-inhibition layer removed. Therefore, clinicians should ensure that the remaining oxygen-inhibition layer is removed to improve the immediate cytocompatibility of restorations.

## Figures and Tables

**Table 1:** Summary of commercially available materials used for the cytotoxicity evaluation.

Product	Manufacturer	Type	Formulation
Filtek™ Z-350XT	3M ESPE, St. Paul, MN, USA	Composite resin	Bis-GMA, UDMA, Bis-EMA, PEGDMA, TEGDMA resins, combination of 20 nm silica filler, 4 to 11 nm zirconia filler, zirconia/silica cluster filler
Adper Scotchbond™	3M ESPE, St. Paul, MN, USA	Bonding resin	Bis-GMA, UDMA, HEMA, glycerol dimethacrylate (GDMA), modified polyacrylic acid, ethanol, water

**Table 2:** Experimental conditions.

Product	Material	Group	Code	Method of Sample Preparation	Curing Condition
Filtek™	Composite resin	C	CU	Uncured	No polymerization
			CD	Direct cured	Direct light curing
Z-350XT			CP	Post-cured	Indirect light curing
			CR	Removed unreacted layer	Indirect light curing
Adper	Bonding resin	B	BU	Uncured	No polymerization
			BD	Direct cured	Direct light curing
Scotchbond™			BP	Post-cured	Indirect light curing
			BR	Removed unreacted layer	Indirect light curing

**Table 3:** Results of the degree of conversion.

Test Materials	Degree of Conversion (%)
CU	0
CD	74.52 ± 2.28
CP	87.59 ± 1.51
CR	95.96 ± 0.07
BU	0
BD	48.71 ± 2.09
BP	61.14 ± 3.48
BR	94.45 ± 0.22

**Table 4:** Results of the agar diffusion test.

Test Materials	Decolorization Index	Lysis Index	Interpretation
Positive control	4	5	Severely cytotoxic
Negative control	0	0	Non-cytotoxic
CU	4	4	Severely cytotoxic
CD	4	5	Severely cytotoxic
CP	3	4	Moderately cytotoxic
CR	2	3	Moderately cytotoxic
BU	4	4	Severely cytotoxic
BD	4	5	Severely cytotoxic
BP	3	4	Moderately cytotoxic
BR	2	3	Moderately cytotoxic
